# En bloc liver and pancreas transplantation after total pancreatectomy with autologous islet transplantation

**DOI:** 10.31373/ejtcm/130187

**Published:** 2020-11-23

**Authors:** Gabriela S. Generette, Piotr J. Bachul, Karolina Golab, Lindsay Basto, Jordan S. Pyda, Peter Borek, Martin Tibudan, Roi Anteby, Laurencia Perea, Michael Charlton, Angelica Perez-Gutierrez, Kumar Jayant, Aaron Lucander, Jeffrey B. Matthews, J. Michael Millis, John Fung, Piotr Witkowski

**Affiliations:** 1 The Transplantation Institute, University of Chicago, USA; 2 Department of Surgery, Beth Israel Deaconess Medical Center, Boston, USA; 3 Department of Surgery, University of Chicago, USA

**Keywords:** Total Pancreatectomy with Autologous Islet Transplantation, TPIAT, liver and pancreas transplantation, chronic pancreatitis

## Abstract

We present a patient with intractable and debilitating pain secondary to chronic pancreatitis who was effectively treated with total pancreatectomy with islet autotransplantation (TPIAT). Islets engrafted into his liver significantly contributed to improved blood glucose control and quality of life. Subsequently, the patient developed alcohol related acute liver failure and en bloc liver and pancreas transplantation was performed to replace the failing liver with engrafted islets. Pancreas transplantation was required to resolve his life-threatening severe hypoglycemic episodes. Herein, we detail an innovative and multidisciplinary management of this complex medical problem.

## Introduction

Treatment of patients presenting with intractable pain due to chronic pancreatitis is complex and challenging [1]. Herein, we present an unusual clinical scenario, in which a patient who had a total pancreatectomy with islet autotransplantation (TPIAT) received a combined en bloc liver and pancreas transplantation due to liver failure. Once the pancreas graft failed, he required subsequent pancreas transplantation due to life threatening severe hypoglycemia. During the clinical course we employed innovative transplantation strategies, which have not yet been universally recognized by the medical community as the standard approach.

## Case report

**Figure 1:**
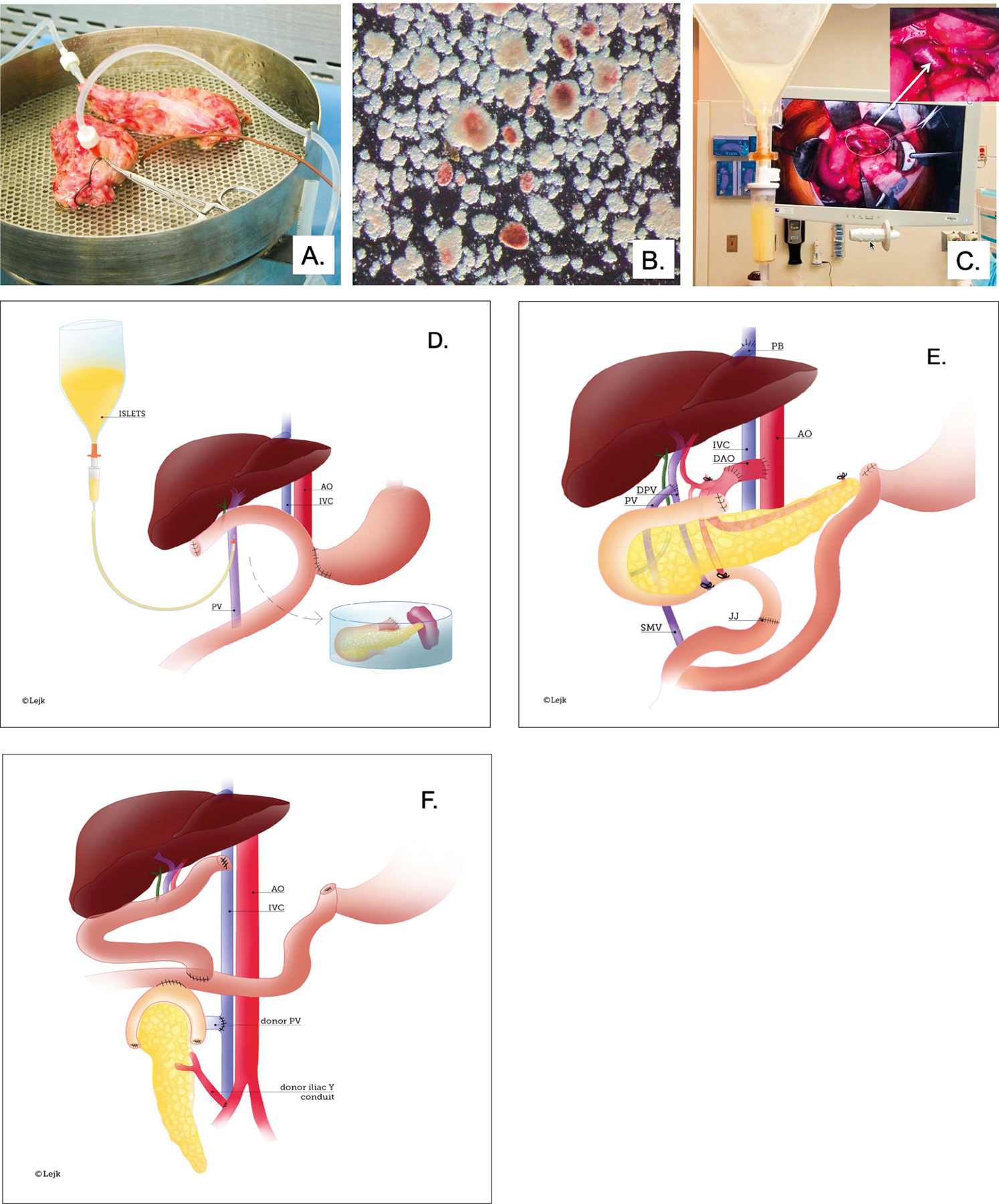
A, B, C. Major steps of islet isolation and islet transplantation Panel A – Total Pancreatectomy. Excised pancreas after total pancreatectomy (TP) was transported to the Good Manufacture Practice (GMP) facility at University of Chicago for processing. The pancreas was divided, and the pancreatic duct cannulated and perfused with collagenase for enzymatic digestion and islet isolation. Panel B – Islet Isolation. After digestion, a mixture of acinar tissue (light brown) and islets (stained with dithizone in red) was collected and the enzyme was washed off. The sample was tested for sterility and endotoxin and was suspended in transplant media in an infusion bag. Panel C – Islet infusion. The portal vein (PV) was cannulated under direct vision and islets were infused intraportally. The white arrow points to the cannulated PV. D, E, F. Illustration of TPIAT and en bloc combined liver pancreas transplantation and solitary pancreas transplantation procedures. Panel D – TPIAT. Schematic demonstrates pancreas excised with duodenum, distal stomach, and spleen and placed on the back-table. Next, pancreatic duct was cannulated for further processing in GMP facility. Once islets were isolated, they were placed in a bag and infused under gravity via the cannulated portal vein. Panel E – En bloc liver and pancreas transplantation. After hepatectomy, the donor hepatic veins were connected to the vena cava using the piggyback technique (PB). The recipient’s portal vein was connected to the side of the portal vein of the donor (PV/DPV). A donor aortic conduit (DAO) was used for the arterial supply to the liver/pancreas graft. Prior to the hepatectomy, the DOA was first anastomosed to the supraceliac recipient aorta (AO) and subsequently, the DOA was connected to a Carrel patch containing the donor celiac trunk and superior mesenteric artery (SMA) during liver/pancreas implantation. Roux-en-Y jejuno-jejunostomy (JJ) restored continuity of the gastrointestinal tract. Panel F – Solitary pancreas transplantation. Schematic demonstrates the position of the transplanted deceased donor pancreas in the right lower abdomen. Arterial blood was provided to the graft via a Y-conduit made of donor iliac arteries. The portal vein of the graft is anastomosed to the distal cava and the duodenum to the side of the jejunum.

**Figure 2:**
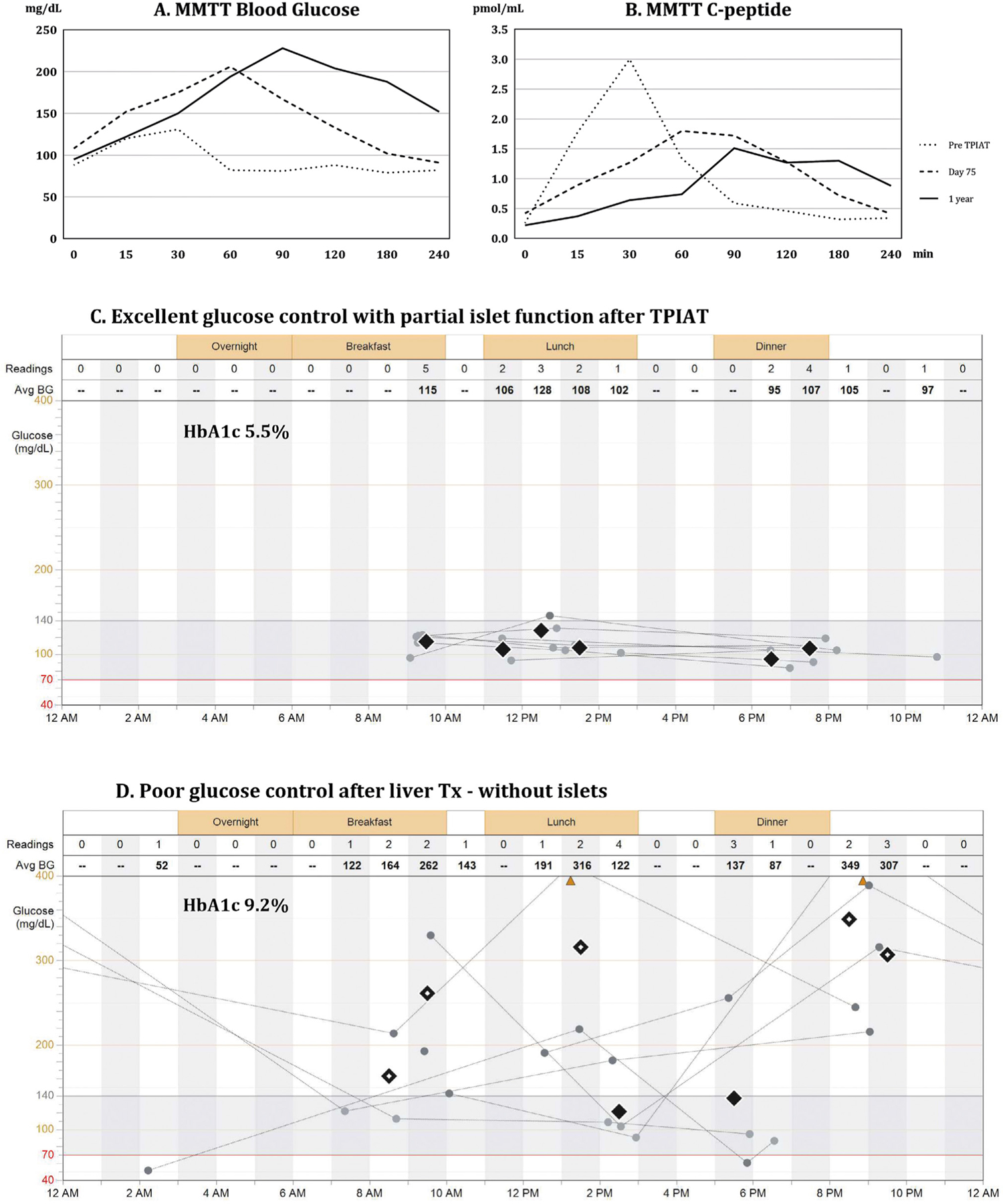
A, B, C, D. Metabolic testing of islet graft function and blood glucose control. Panel A presents blood glucose levels, whereas Panel B presents serum c-peptide levels during mixed meal tolerance test (MMTT), which was performed prior to the procedure (dotted line), at postoperative day 75 (dashed line), and at 1 year (continuous line) after total pancreatectomy with islet autotransplantation (TPIAT). Prior to surgery, islets controlled blood glucose during the mixed meal challenge; fasting blood glucose was 100 mg/dL, peaked at 120 mg/dL around 30 minutes after consumption of mixed meal, and dropped to normal values afterwards. This was demonstrated by levels of serum c-peptide, which corresponds to endogenous insulin released from islets (Panel A, B, dotted lines). After TPIAT, insulin released from the islet autograft, as represented by serum c-peptide (Panel B, dashed and continuous line), prevented a further rise of blood glucose above 230 mg/dL during the MMTT (Panel A, dashed and continuous line). The test confirmed partial islet graft function by demonstrating a clinically significant insulin release from the islet graft and the need for exogenous support for optimal glucose control. Panel C presents daily trends of blood glucose recorded over a period of one week in the patient after TPIAT with partial islet graft function, allowing for optimal glucose control (HbA1c 5.5%). Most of the time blood glucose remained within the normal range of 70–150 mg/dL, without episodes of hyper- or hypoglycemia. Panel D presents extremely poor glucose control in the same patient following liver transplantation with hepatectomy of the liver, which contained the islet autograft. The patient struggled with insulin dose adjustments despite using an insulin pump, glucose monitoring system, and intensive diabetic care. He had persistent hyperglycemia (HbA1c 9.2%) with episodes of both severe hyperglycemia (> 400 mg/dL) and hypoglycemia (< 54 mg/dL) with loss of consciousness and seizures.

A 38-year-old, non-diabetic male with chronic pancreatitis and heterozygous CFTR (cystic fibrosis transmembrane conductance regulator) gene mutation (p.R553c.1657C > T) was referred for intractable and debilitating pain persisting over five years despite multiple endoscopic interventions. The clinical course was complicated by malnutrition requiring enteral tube feeding. He had a BMI of 34 and a family history of non-alcoholic fatty liver disease (NAFLD). Computer tomography revealed an atrophic pancreas with calcifications and a non-dilated pancreatic duct. The patient underwent TPIAT: 152,000 islet equivalents suspended in 16 mL of tissue were infused into his portal vein (Figure 1A–D) [1]. The patient was completely weaned off opioid analgesics over the next few months and returned to full professional activity. Blood glucose was successfully managed with approximately 17 units of insulin per day and hemoglobin A1c (HbA1c ) was 5.5% at 1 year follow up (Figure 2). Mild transaminase elevation was noted and attributed to NAFLD. His alcohol consumption during this period was approximately two drinks per day, three days per week.

Two years later, the patient was urgently hospitalized due to decompensated liver cirrhosis manifested by ascites, encephalopathy and hepatorenal syndrome. He had a MELD (Model For End-Stage Liver Disease) score of 42 (serum creatinine 2.7 mg/dL, total bilirubin 18.6 mg/dL, international normalized ratio 3.9). A liver biopsy demonstrated steatohepatitis with extensive fibrosis. The patient was thought to be ineligible for liver transplantation at a local center due to excessive alcohol use. Hospice care was initially advised; however, he was ultimately transferred to our center for re-evaluation.

We determined the patient to be an eligible candidate for liver transplantation consistent with recent progressive practice [2]. We reasoned that his acute liver failure was the initial presentation of liver disease and did not represent a failure of abstinence from alcohol - a traditional exclusion criterion for liver transplantation. Furthermore, in addition to alcohol consumption, his liver failure could have been multifactorial, reflecting the progression of nonalcoholic steatohepatitis, and be related to multiple metabolic derangements associated with his prior pancreatic disease. He underwent an emergent en bloc liver and pancreas transplantation to compensate for both the impending loss of liver metabolic function as well as the sacrifice of the islet autotransplant during the explant of the native liver (Figure 1E). The cold ischemia time was six hours and we employed a porto-systemic veno-venous bypass for the transplant. The revascularization of the en bloc liver-pancreas graft was performed via the piggyback technique, with a porto-portal vein anastomosis and an aortic conduit to the recipient supraceliac aorta (Figure 1E). The small bowel was anastomosed to the side of the first loop of the donor jejunum. Due to significant hemorrhage from a friable aortic conduit anastomosis, pancreas graft warm ischemia was extended to 2 hours and the patient required extensive intra-operative vasopressor support and resuscitation with 23 liters of blood products. On post-operative day one, the patient developed hemodynamic instability and required re-exploration. The pancreas graft was found to be thrombosed and it was excised. The remainder of the post-operative course was uneventful, and the patient was discharged to a rehabilitation facility on post-operative day ten with steroid free immunosuppression.

Over the next several months, the liver graft function was excellent, but blood glucose control remained poor despite intensive diabetic care, including the use of MINIMED 760G insulin pump, 630G system with SmartGuard® technology and Contour Next Link 2.4 glucose monitor. The HbA1c increased from 5.7% to 9.2%, and the patient experienced severe hypoglycemic episodes leading to seizures and an urgent hospitalization (Figure 2D). His quality of life was severely compromised with the progression of anxiety and depression, leading to a suicide attempt. Eventually, ten months following en bloc liver and pancreas transplantation, the patient underwent uneventful solitary pancreas transplantation in the right iliac fossa with enteric drainage (Figure 1F). Excellent glucose control was immediately restored and his quality of life improved. The patient has remained compliant with medical therapy for over three years now. Liver and pancreas graft function have remained stable with HbA1c maintained below 5% without exogenous insulin. Anxiety and depression were medically controlled and he remains abstinent from alcohol.

## Discussion

Total pancreatectomy (TP) is an effective treatment for intractable pain due to chronic pancreatitis in properly selected patients [1, 3]. Progression of chronic pancreatitis is often inexorable, particularly when associated with underlying genetic mutations, and these patients are often the best candidates for this approach [4]. Indeed, our patient had benefited greatly from TP with complete resolution of pancreatic-type abdominal pain and opioid dependency permitting a return to full professional activity.

Since TP induces type 3c diabetes, simultaneous islet autotransplantation (IAT) is indicated to improve postoperative glycemic control. Despite IAT, most patients (60–80%) require insulin supplementation and hence some physicians and insurance carriers are hesitant to pursue IAT [3–5]. However, IAT reduces the amount of insulin supplementation required, and more importantly, restores a degree of counterregulation which protects from debilitating severe hypoglycemic episodes, as evidenced by our patient [3, 5–7]. This improves glucose control and helps to maintain normal HbA1c values [6]. The benefit of IAT extends beyond the 30% of patients who achieve insulin independence to an additional 50–58% who retain partial islet function (requiring some insulin support) [3, 5]. Overall, quality of life is notably improved in patients after TPIAT [3, 5, 7]. Contemporary evidence in the literature enormously values TPIAT and makes it essentially unethical to perform TP without IAT in otherwise suitable candidates. Furthermore, as immunosuppression is not required following the procedure, the additional risk of IAT is minimal and the rate of post-operative portal vein thrombosis is low and can be minimized with judicious post-operative anticoagulation [8]. Recognizing these advantages, the majority of commercial insurance carriers in the United States and in several European countries should consider reimbursing IAT. Hopefully, the multicenter TPIAT study, which is currently underway, will provide further evidence to change the position of the remaining insurance carriers (notably the Centers for Medicare and Medicaid Services) and clinicians who have not yet recognized the benefits of IAT [9].

Our patient benefited from partial islet function after IAT by achieving good glycemic control with the help of an insulin pump (HbA1c 5.5%) and improved pain control. However, the hepatectomy of the failing liver led to the loss of the islet autograft. Advanced diabetic care was needed but not able to provide sufficient glycemic control and the patient experienced extreme blood glucose liability and hypoglycemic seizures. Hence, our case provides invaluable evidence regarding the clinical benefit of even partial islet function especially considering that the comparison is derived from the same patient who received the same advanced medical regimen.

Anticipating challenges in the management of diabetes after the excision of the failing liver with engrafted autologous islets, we performed pancreas and liver transplantation at the same time. Standard heterotopic pancreas transplantation in the pelvis is often challenging due to cardiovascular instability and congestion of the bowel during liver transplantation with extended overall operative time. Therefore, we decided to perform liver/pancreas implantation en bloc, which requires only the addition of an arterial conduit and one bowel anastomosis compared to liver transplantation alone [10]. Moreover, the liver confers a known immunoprotective effect for the same-donor organs transplanted simultaneously [11].

Even in completely stable patients undergoing solitary pancreas transplantation, the rate of pancreas thrombosis is 10% and results from increased vascular resistance coinciding with hemodynamic instability or reperfusion injury [6]. Timely excision of the necrotized pancreatic graft was critical for the intra-operative stabilization of our patient and his subsequent recovery. The re-operation only required an additional hepatico-jejunostomy, which was completed without interrupting any blood supply to the liver graft. Nevertheless, in future cases of patient instability, it would be judicious to perform the liver anastomosis first and defer the pancreas implantation until the hemodynamics improve. However, should the patient remain hemodynamically unstable or have a persistent vasopressor requirement, the pancreas graft can be implanted in a backup recipient.

Subsequent solitary pancreas transplantation proceeded uneventfully in our patient. Its benefits are well described for T1DM patients already on immunosuppression and in those with life-threatening episodes of severe hypoglycemia despite appropriate endocrine management [6, 12]. Our patient’s clinical course highlights the continued advantage of pancreas and islet transplantation in diabetic patients who suffer from problematic hypoglycemia despite being on modern insulin delivery systems and glucose monitoring.

This case also supports the recent re-examination of a long-held paradigm of excluding active alcoholics from liver transplantation. Our patient was initially disqualified from liver transplantation at another center due to suspected alcohol abuse. Donor organ scarcity and high rates of recurrent liver failure in active alcoholics have led to a six-month sobriety requirement prior to consideration for transplantation. While this might be a reasonable approach for patients with chronic liver disease, it seems inappropriate if acute liver decompensation is the first manifestation of alcohol-related disease because such patients never had a chance to obtain proper treatment for alcoholism and meet the 6-month sobriety requirement. Recently published relapse rates are low and our patient’s recovery supports the benefit of more nuanced criteria for liver transplantation in active alcoholics [13].

The etiology of the liver cirrhosis in our patient was multifactorial, including obesity, suspected NAFLD, and CFTR mutation carrier status compromising pancreatic function which collectively contributed to liver failure [14]. Our patient suffered from steatorrhea despite enzyme supplementation, before and after TPIAT, which may have further contributed to steatohepatitis and its progression to cirrhosis as a consequence of choline deficiency, diminished phospholipid synthesis and failure of fat transport and oxidation [15]. Additionally, the patient had a history of alcohol consumption, which although moderate, may have exacerbated liver damage and led to progression of cirrhosis [16].

In summary, this case illustrates the numerous advantages of IAT and underscores its role in minimizing serious hypoglycemic events despite only partial islet graft function and persistence of post-operative insulin dependence. Furthermore, it demonstrates the value of liver transplantation in patients with acute liver failure secondary to alcohol. Lastly, it highlights the lifesaving role of pancreas transplantation in diabetic patients with problematic hypoglycemia and no remaining beta cell function.
